# Experimental Investigation of Curvature Effects on the Bond–Slip Behavior of ECC–Steel Tube Interfaces

**DOI:** 10.3390/ma19194114

**Published:** 2026-09-26

**Authors:** Qian Li, Ruming Ban, Huan Zhao, Xi Li, Juncheng Bai

**Affiliations:** 1 School of Intelligent Construction, Qingdao Binhai University, Qingdao 266555, China; liqianchiping@outlook.com (Q.L.); junchbai@163.com (J.B.); 2 School of Civil Engineering, Qingdao University of Technology, Qingdao 266520, China; banruming@stu.qut.edu.cn (R.B.); zhaohuanfun6@outlook.com (H.Z.)

**Keywords:** ECC–steel tube composite member, steel–tube curvature, interfacial bond–slip behavior, push-out test, bond–slip model

## Abstract

To quantify the effect of steel tube curvature on bond–slip behavior at the ECC–steel tube interface, 12 push-out specimens were tested in four groups. Steel–tube curvature was varied as *K* = 0, 0.01, 0.012, and 0.02, while the ECC cover thickness (20 mm), tube wall thickness (6 mm), and bond length were held constant. Monotonic push-out tests were used to obtain interfacial bond stress–slip curves and to assess failure modes, bond strength, and characteristic slip. A curvature-dependent piecewise bond–slip model was then calibrated to the test data. Within the investigated range, increasing curvature improved the measured interfacial response. As *K* increased from 0 to 0.02, the peak bond stress rose from 0.236 to 0.644 MPa (172.9%), while the characteristic slip at the onset of the residual stage increased from 2.696 to 4.630 mm (71.7%). The proposed model reproduced the measured curves with coefficients of determination R^2^ ≥ 0.96 and an average fitting error below 4.1%. These findings indicate that steel–tube curvature can enhance interfacial load transfer and deformation capacity within the tested parameter range and provide a basis for interface design and numerical modeling of ECC–steel tube composite members.

## 1. Introduction

Growing demands for load-carrying capacity, ductility, and durability in bridges, high-rise buildings, and prefabricated construction have accelerated the development of high-performance composite systems. Steel tube-engineered cementitious composite (ECC) members combine the high load-carrying capacity and confinement provided by steel tubes with the high ductility, crack resistance, and damage tolerance of ECC. This complementarity gives these systems considerable potential for bridge structures, building components, and seismic retrofitting applications [1,2,3]. Under external loading, interfacial bond enables load transfer and deformation compatibility between the steel tube and the ECC matrix. This bond governs composite action and strongly influences member capacity, ductility, stiffness degradation, and failure mode [4,5]. Consequently, a systematic understanding of steel tube–ECC interfacial behavior and its governing parameters is essential for elucidating composite action, developing constitutive models, and supporting engineering applications. Accordingly, inadequate interfacial bond may prevent the steel tube and ECC matrix from fully mobilizing their complementary mechanical advantages, even when the two constituent materials retain sufficient individual strength.

Experimental and theoretical studies have investigated bond behavior at steel tube–ECC interfaces and produced a substantial body of evidence. Existing experimental work has primarily used push-out tests [6] and pull-out tests [7] to characterize load transfer, bond–slip response, and failure modes. Numerical studies have more commonly examined steel tubes combined with other high-performance cementitious materials, such as UHTCC [8] or UHPC [9]; their interface modeling strategies provide a useful basis for steel tube–ECC numerical analysis. Previous studies have indicated that load transfer at the interface is governed by adhesion, mechanical interlock, and frictional resistance [10,11,12]. Interfacial bond capacity and bond–slip response depend on the steel tube surface condition, interface treatment, ECC strength, fiber volume fraction, and bond length [13,14,15,16]. Accordingly, surface modification, interface treatment, and mixture optimization can enhance bond strength and composite action. The available literature, however, has focused primarily on material parameters and interface treatments; the effect of steel tube geometry, particularly curvature, remains comparatively underexplored. The relative contributions of adhesion, frictional resistance, and mechanical interlock evolve as interfacial slip develops. Adhesion is considered to play a major role in the initial stage, whereas frictional resistance and mechanical interlock become increasingly important after debonding. This evolution provides a mechanistic basis for examining how steel tube curvature affects the bond–slip response.

As a key cross-sectional geometric parameter, steel tube curvature governs the contact geometry at the steel tube–ECC interface and may alter normal contact pressure, contact area, and stress distribution [17], thereby affecting interfacial load transfer [18] and bond–slip response. From an interface mechanics perspective, increasing curvature may mobilize greater frictional resistance and geometric interlock [19,20]. Conversely, excessive curvature may induce local stress concentrations and reduce interface stability [21,22]. Existing studies, however, have focused mainly on material properties [23], interface treatments [24], and surface morphology [25]. The quantitative relationship between steel tube curvature and interfacial bond response therefore remains insufficiently understood, as does the mechanism by which curvature affects stress transfer and slip evolution. Experimental evidence and an interface mechanics interpretation are needed to address this gap.

Substantial progress has also been made in developing bond–slip constitutive models for steel tube–concrete interfaces. Most models are calibrated from experimental data to relate interfacial bond stress to slip [26,27,28]. More recent studies have incorporated parameters such as cross-sectional geometry and cover thickness to improve predictive capability and engineering applicability [29,30,31]. For steel tube–ECC interfaces, however, the available constitutive formulations remain limited. In particular, the curvature effect has not been quantified systematically or incorporated into an interface model. A constitutive relationship that does not explicitly account for curvature has limited ability to represent systematic differences in bond–slip response among interfaces with different contact geometries under otherwise comparable test conditions. Curvature was therefore incorporated into the characteristic parameters of the bond–slip relationship to represent the response variation observed within the present test matrix.

To address these gaps, this study used monotonic push-out tests to examine the effect of steel tube curvature on interfacial bond behavior. Twelve composite specimens were divided among four curvature groups (*K* = 0, 0.01, 0.012, and 0.02), with three nominally identical specimens in each group. Observed failure modes, complete bond stress–slip curves, and characteristic response parameters were considered together to compare bond strength, slip evolution, and load transfer behavior across the four curvature levels. Compared with existing studies that have mainly focused on material properties, steel surface conditions, interface treatments, and bond length, this study focuses on steel tube curvature as a cross-sectional geometric parameter and quantitatively investigates its effects on the peak bond stress, post-peak degradation, residual bond stress, and characteristic slip of the ECC–steel tube interface. Because the fitted relationships were derived from the same test dataset, they should be regarded as calibration relationships within the investigated curvature range rather than as independently validated predictive equations. The resulting test data and constitutive formulation provide a basis for interface design and numerical modeling of ECC–steel tube composite members.

## 2. Materials and Methods

### 2.1. Specimen Design

Four groups of ECC-encased steel tube push-out specimens were fabricated, with three nominally identical specimens in each group. Steel tube curvature *K* was the primary experimental variable and was set to 0, 0.01, 0.012, or 0.02. In this study, the curvature of the circular steel tube surface is defined as K=1/R=2/D, where *R* and *D* denote the outer radius and outer diameter of the circular steel tube, respectively. When the geometric dimensions are expressed in millimeters, the unit of *K* is mm^−1^. A larger value of *K* corresponds to a smaller tube radius and a more pronounced curvature of the interface. Accordingly, the variation in tube diameter is inherent to the prescribed curvature levels rather than an independent geometric variable. For the square steel tube reference specimens, the principal steel–ECC contact surfaces are planar and may be regarded as having an infinitely large radius of curvature; therefore, their curvature is defined as *K* = 0. The curvature levels were selected primarily based on standardized steel tube dimensions and geometric considerations. The circular steel tubes had outer diameters of 219, 159, and 108 mm, which are standard pipe sizes specified in GB/T 28708-2012 [32] and are consistent with the approximately 100–200 mm diameter range commonly adopted in existing experimental studies on concrete-filled steel tubular structures. Based on the curvature definition K=2/D, these specimens were represented by the nominal curvature levels of 0.01, 0.012, and 0.02 mm^−1^, respectively. The selected dimensions provide progressively increasing and sufficiently distinct interface curvatures while retaining standardized steel tube sizes. Other major geometric parameters, material properties, and loading conditions were kept consistent among the specimen groups to minimize potential confounding effects. The *K* = 0 specimens used square steel tubes as the reference group. The ECC cover thickness, steel tube wall thickness, specimen height, and effective bond length were maintained at 20, 6, 319, and 259 mm, respectively. No studs, grooves, or other shear connectors were provided; the steel surface in contact with ECC was derusted and polished. Specimens were cast in custom wooden molds and cured for 28 days under standard conditions before testing. Table 1 lists the specimen parameters, and Figure 1 illustrates the geometry and dimensions. Representative photographs of the specimen fabrication process and the specimens before testing are shown in Figure 2. Keeping these parameters constant reduced interference from cover thickness, wall thickness, and bond length, thereby allowing the measured differences among groups to be attributed primarily to the prescribed curvature.

### 2.2. Material Properties

The specimens comprised PP fiber-reinforced ECC and Q235 steel tubes. Table 2 gives the ECC mass proportions and PP fiber properties, and Table 3 summarizes the steel tube properties. The tube wall thickness was 6 mm, and the cross-sectional size varied with the prescribed curvature. The steel met the requirements of GB/T 700–2006 [33], Carbon Structural Steels. The tube strength and wall thickness were selected to avoid premature global failure during push-out loading so that the measured response would be governed primarily by the steel tube–ECC interface.

### 2.3. Loading and Measurement

Interfacial bond behavior was evaluated using a Hualong 700 t compression–shear testing machine (Shanghai Hualong Test Instruments Corporation, Shanghai, China); Figure 3 shows the test setup. A 20 mm thick steel bearing plate, whose opening matched the steel tube cross-section, was placed beneath each specimen to support the ECC while allowing the tube to be pushed through. A downward axial load was applied to the protruding steel tube. Displacement-controlled loading was imposed at 0.2 mm/min, and force and displacement were recorded continuously. Loading was terminated when the measured free-end slip reached 10 mm. This protocol captured the response from initial adhesion through progressive slip to the residual friction stage while limiting secondary damage to the specimen. The matched opening in the bearing plate allowed the ECC matrix to be supported while permitting the steel tube to move through the opening, thereby minimizing direct restraint during push-out loading. The recorded load and free-end displacement were consequently used to characterize the progressive interfacial bond–slip response under consistent test conditions.

## 3. Experimental Results and Discussion

### 3.1. Failure Modes and Interfacial Mechanism

All specimens remained globally intact during the push-out tests. No visible cracking, spalling, or peeling occurred on the outer ECC surface, and no matrix crushing, local fragmentation, or steel tube buckling was observed. Failure was localized at the steel tube–ECC interface. As displacement increased, adhesive bond deteriorated and interfacial slip developed, while frictional resistance and geometric interlock progressively governed load transfer. At large slips, the response transitioned to a residual friction stage rather than complete loss of load transfer. Figure 4 shows the post-test failure appearances, and the following paragraphs compare the observed responses among the four curvature groups.

Post-test inspection showed systematic differences in the interfacial traces and load degradation behavior among the four curvature groups. The change from limited interfacial traces at *K* = 0 to more evident frictional traces at higher curvature was consistent with a shift in the relative contributions of adhesion, frictional resistance, and geometric interlock as the interface geometry changed. These observations were interpreted together with the measured post-peak response and were not regarded as direct measurements of local contact pressure or stress distribution.

Representative details of the steel–ECC interface after the push-out tests are shown in Figure 5. Clear frictional traces and local surface damage can be observed at the interface, indicating that interfacial sliding and frictional interaction occurred during the push-out process.

For the reference specimens with square tubes (*K* = 0), the load increased steadily during the initial stage and then decreased sharply after the peak, accompanied by a rapid increase in relative slip. The exposed ECC interface remained intact, with little evidence of frictional scratching or local bearing. The response was therefore characterized as predominantly interfacial shear slip. Because the flat faces provided limited geometric interlock, the loss of adhesion after the peak was not sufficiently compensated for by local bearing or sustained frictional resistance, resulting in rapid sliding and comparatively limited residual resistance.

At *K* = 0.01, the post-peak load decreased more gradually than in the reference specimens. Interfacial slip was accompanied by progressive frictional sliding and limited geometric interlock, which helped sustain load transfer during the descending branch. Light frictional scratches were observed on the ECC interface, while no spalling, delamination, or cracking occurred. The failure mode was therefore characterized as stable frictional sliding with limited geometric interlock.

At *K* = 0.012, the post-peak decline was gentler than that observed at *K* = 0.01, and the interface maintained comparatively stable bond resistance during slip. The curved contact geometry may have enhanced local bearing and increased the contributions of frictional resistance and geometric interlock, thereby promoting a more progressive slip response. No abrupt debonding or visible ECC damage was observed. The failure mode was therefore characterized by progressive interfacial slip associated with the combined action of frictional resistance and geometric interlock.

Specimens with *K* = 0.02 exhibited the highest bond resistance and the most gradual post-peak response within the tested range. The interface maintained comparatively stable resistance as slip developed, and pronounced frictional traces were observed on the exposed interface. These observations are consistent with sustained frictional resistance and geometric interlock. The specimens therefore exhibited stable, progressive interfacial slip rather than abrupt debonding.

Across all groups, failure remained localized at the interface, whereas the ECC matrix and steel tube retained their overall integrity. Increasing displacement caused loss of adhesion, followed by progressive frictional sliding and geometric interlock; residual resistance remained at large slip. The common loading rate and termination criterion enabled consistent comparison of the bond stress–slip response across curvature groups. These data form the basis for the quantitative analysis that follows.

Based on the above experimental observations, a simplified mechanical interpretation is introduced to explain the possible influence of steel tube curvature on the interfacial load transfer mechanism. For a thin-walled circular steel tube, the radial stiffness can be expressed as:

(1) $$k_{s}=\frac{E_{s}t}{R^{2}}=E_{s}tK^{2}$$

where *E_s_* is the elastic modulus of steel, *t* is the steel tube wall thickness, *R* is the interface radius, and *K =* 1*/R* is the interface curvature. Under comparable local radial deformation *u_r_*, the normal contact pressure may be written as:

(2) $$p_{n}=k_{s}u_{r}$$

Thus, increasing curvature may enhance the radial restraint and normal contact pressure at the steel–ECC interface. According to:

(3) $$\tau_{f}=\mu p_{n}$$

where *τ_f_* is the interfacial frictional shear stress and *μ* is the friction coefficient, the increased contact pressure may strengthen frictional resistance. Stronger normal restraint may also help maintain the engagement of surface asperities, thereby sustaining local mechanical interlock during slip. The proposed mechanism is schematically illustrated in Figure 6.

### 3.2. Bond Stress–Slip Response

The average interfacial bond stress, *τ*, was calculated as follows:

(4) $$\tau =\frac{P}{LC}$$

where *P* is the measured interfacial load, *L* is the effective bond length, and *C* is the steel tube perimeter at the interface. For the circular steel tube specimens, the interface perimeter *C* was calculated as π*D*; therefore, the nominal effective bond area was determined from the cylindrical interface geometry as *A_b_* = π*DL*, rather than from the projected planar area. The bond stress was normalized by the unit interface area, thereby reducing the direct influence of different interface areas on the comparison among specimens. In addition, an appropriate amount of expansive agent was incorporated into the ECC to compensate for shrinkage and minimize shrinkage-induced interface separation and debonding.

The measured load and slip were converted to bond stress–slip curves for each specimen group (Figure 7). The characteristic parameters listed in Table 4 were identified using the idealized response shown in Figure 8. The idealized characteristic points separate the response into the rising stage, the initial post-peak decrease, and the residual stage. This representation permits the effects of curvature on strength, deformation, and post-peak stability to be discussed using the same set of parameters for every group.

Figure 7 and Table 4 show that, within the tested range, increasing steel tube curvature improved the interfacial response. The peak bond stress for specimens with *K* = 0.02 was 0.644 MPa, compared with 0.236 MPa for the square tube reference specimens (*K* = 0), an increase of 172.9%. The corresponding peak slip increased from 0.600 mm at *K* = 0 to 1.375 mm at *K* = 0.02, indicating that greater slip was required to mobilize peak bond resistance as curvature increased. Curvature also moderated the post-peak decline. The reference specimens (*K* = 0) exhibited a steep descending branch, whereas specimens with *K* = 0.01, *K* = 0.012, and *K* = 0.02 showed progressively gentler degradation. After the post-peak decline, each curve approached a residual plateau. For *K* = 0.02, the degraded stage characteristic stress was 0.400 MPa. At the residual stage, bond stress increased from 0.113 MPa for *K* = 0 to 0.317 MPa for *K* = 0.02, or approximately 2.81 times the reference value. The slip corresponding to the onset of the residual stage also increased from 2.696 mm at *K* = 0 to 4.630 mm at *K* = 0.02, representing a 71.7% increase. These results indicate that the higher-curvature interface sustained progressive load transfer over a larger relative displacement before reaching the residual regime.

To quantify the specimen-to-specimen variability, the standard deviations (*SD*s) of the characteristic bond stresses and slips were calculated for each curvature group, as summarized in Table 5.

The results show that the degree of scatter varied among the characteristic parameters and curvature groups. Considering the limited number of replicate specimens, the *SD* values were used to quantify the repeatability and dispersion of the experimental responses rather than to establish statistical significance.

The increase in bond resistance with increasing curvature may be associated with the more pronounced curved interface geometry. As the tube radius decreases, the curved interface may provide stronger geometric restraint and help maintain steel–ECC contact during interfacial slip. Consequently, frictional resistance and local geometric interlock may remain effective after the degradation of adhesive bonding, resulting in a higher peak bond stress, a more gradual post-peak degradation, and a higher residual bond resistance. This interpretation is consistent with the more pronounced interfacial friction traces and the enhanced post-peak resistance observed for the higher-curvature specimens.

Overall, curvature was an influential geometric parameter within the test matrix. Relative to the square tube reference, greater curvature increased peak and residual bond stress, moderated post-peak degradation, and promoted more stable load transfer at large slip. The combined contributions of frictional resistance and geometric interlock provide a plausible explanation for the higher strength and more ductile slip response observed in the curved tube specimens. These trends should, however, be interpreted within the investigated curvature range. The present data demonstrate improved interfacial performance over this range but do not establish that the response will continue to improve monotonically beyond the tested geometries or under different material and loading conditions.

### 3.3. Analysis of Curvature Parameters of Steel Tubes

To characterize the relationship between steel tube curvature (*K*) and interfacial bond response, the group-level peak, degraded stage, and residual bond stresses were compared at a constant ECC cover thickness (Figure 9). Gray relational analysis was used as a descriptive measure of trend similarity between the curvature sequence and the corresponding bond–stress sequences. The calculated relational grades were interpreted comparatively rather than according to a universal classification threshold and were not treated as measures of statistical significance. The calculation procedure is summarized below.

The peak bond stress sequence was selected as the reference sequence for the gray relational analysis $X_{0}=\left\{x_{0}\left(1\right),x_{0}\left(2\right),\ldots ,x_{0}\left(4\right)\right\}$. Here, *n* = 4 denotes the number of curvature groups. The curvature sequence was used as the comparison sequence $X_{1}=\left\{x_{1}\left(1\right),x_{1}\left(2\right),\ldots ,x_{1}\left(4\right)\right\}$. This analysis evaluates the association between curvature and peak bond stress over the investigated parameter range.

Because curvature and bond stress have different dimensions and magnitudes, their unscaled numerical differences are not directly comparable. Initial value normalization was therefore applied to preserve the relative trends while rendering both sequences dimensionless:

(5) $$x_{i}^{\prime }\left(k\right)=\frac{x_{i}\left(k\right)}{x_{i}\left(1\right)}\;\left(i=0,1;k=1,2,\ldots ,n\right)$$

where $x_{i}\left(k\right)$ denotes the data for the *k*-th curvature group in the original sequence; $x_{i}^{\prime }\left(k\right)$ is the corresponding normalized value; and $x_{i}\left(1\right)$ is the first value of that sequence.

The absolute difference between the corresponding normalized reference and comparison values was then calculated at each curvature level:

(6) $$\Delta_{i}=\left|x_{0}^{\prime }\left(k\right)-x_{i}^{\prime }\left(k\right)\right|\;\left(i=1;k=1,2,\ldots ,n\right)$$

The minimum and maximum absolute differences across the four curvature groups are denoted by $min_{i,k}\Delta_{i}(k)$ and $max_{i,k}\Delta_{i}(k)$, respectively, and are used to calculate the local relational coefficients.

The relational coefficient quantifies the local similarity between the normalized curvature and bond stress sequences at each data point:

(7) $$\xi_{i}\left(k\right)=\frac{min_{i,k}\Delta_{i}(k)+0.5\cdot max_{i,k}\Delta_{i}(k)}{\Delta_{i}(k)+0.5\cdot max_{i,k}\Delta_{i}(k)}$$

The distinguishing coefficient was set to ξ=0.5, a commonly adopted value that balances sensitivity and stability. A coefficient closer to 1 indicates greater local similarity between the normalized sequences.

The arithmetic mean of the four local relational coefficients was taken as the gray relational grade:

(8) $$r_{i}=\frac{1}{n}\sum_{k=1}^{n}\xi_{i}\left(k\right)\;\left(i=1\right)$$

where $\xi_{i}\left(k\right)$ denotes the gray relational coefficient between the reference sequence and the *i* -th comparison sequence at the *k* -th data point, and $r_{i}$ denotes the corresponding gray relational grade. In this study, only one comparison sequence was considered; therefore, *i* = 1, and *n* = 4 represents the four curvature groups. After completing the calculations based on the measured test data, a gray relational grade of 0.75 was obtained between steel tube curvature and peak interfacial bond stress. This result indicates a clear similarity between the two sequences within the investigated range and is consistent with the measured increase in peak bond stress as curvature increased. Under otherwise controlled test conditions, steel tube curvature can therefore be regarded as an influential geometric parameter affecting the interfacial bond response of ECC–steel tube composite specimens.

Empirical relationships between curvature and the characteristic bond stress and slip parameters were fitted to the four group-level data points as follows:

(9) $$\tau_{u}=0.188414+19.74631K$$

(10) $$\tau_{d}=0.13969+12.52956K$$

(11) $$\tau_{r}=0.099172+10.07882K$$

(12) $$S_{u}=0.543759+38.40394K$$

(13) $$S_{d}=0.709241+74.73892K$$

(14) $$S_{r}=2.390379+92.63054K$$

where *K* denotes the steel tube curvature; *τ_u_*, *τ_d_*, and *τ_r_* are in MPa; *S_u_*, *S_d_*, and *S_r_* are in mm.

Substitution of the curvature values into Equations (9)–(14) yielded the calculated bond stress and slip parameters shown in Table 6; Table 7 gives the corresponding calculated-to-measured ratios.

The column-wise mean calculated-to-measured ratios in Table 7 are 1.0020, 0.9983, 0.9948, 0.9995, 1.0385, and 1.0060, respectively. These values indicate satisfactory agreement for the calibration data. The proposed relationships were calibrated using the four curvature levels investigated in this study; therefore, their applicability is limited to the tested curvature range, and further experimental verification is required before extending them to curvature values outside this range or to different material and geometric conditions.

In the idealized response shown in Figure 8, points A(*S_u_*,*τ_u_*), B(*S_d_*,*τ_d_*), and C(*S_r_*,*τ_r_*) are the characteristic points of the bond stress–slip curve. Here, *S_u_*, *S_d_*, and *S_r_* are the slip values corresponding to *τ_u_*, *τ_d_*, and *τ_r_*, respectively. The resulting piecewise bond stress–slip relationship is given by Equation (15).

(15) $$\tau =\left\{\begin{array}{l} \frac{\tau_{u}}{S_{u}}\cdot S,0\leq S<S_{u}\\ \tau_{u}+\frac{\tau_{d}-\tau_{u}}{S_{d}-S_{u}}\cdot (S-S_{u}),S_{u}\leq S<S_{d}\\ \frac{S\cdot \left[\tau_{d}\tau_{r}\left(S_{d}-S_{r}\right)\right]}{(S_{d}\tau_{r}-S_{r}\tau_{d})S+S_{d}S_{r}\left(\tau_{d}-\tau_{r}\right)},S_{d}\leq S<S_{r}\\ \tau_{r},S\geq S_{r} \end{array}\right.$$

Substituting the fitted parameters into Equation (15) yielded the calculated bond stress–slip curves, which are compared with the experimental curves in Figure 10.

The calculated curves show satisfactory agreement with the measured responses in terms of overall curve shape. In particular, the proposed model reproduces the increase in interfacial bond stress, the development of slip, and the post-peak degradation trend. The coefficient of determination, R^2^, is not less than 0.96, and the average error is below 4.1%. These results indicate that the proposed curvature-dependent relationships satisfactorily capture the variation of the key bond–slip parameters with steel tube curvature within the investigated range.

## 4. Conclusions

To investigate the effects of steel tube curvature on the bond–slip behavior of ECC–steel tube interfaces, a series of static push-out tests was conducted using steel tube curvature as the sole control variable. Under consistent specimen parameters, fabrication procedures, and loading protocols, the failure modes and interfacial mechanical responses of specimens with different curvature levels were systematically examined. The effects of curvature on peak interfacial bond strength, stress degradation behavior, and residual bond–slip behavior were analyzed. Based on the experimental results, a corresponding interfacial bond–slip constitutive model was established. The main conclusions of this study are summarized as follows:

1. Within the tested range, increasing steel tube curvature changed the interfacial response from abrupt post-peak slip toward more stable and progressive sliding. The square tube specimens (*K* = 0) exhibited limited geometric interlock, and their load transfer depended mainly on adhesion and frictional resistance. In comparison, the curved tube specimens showed more evident frictional engagement and geometric interlock, while the ECC remained visibly intact and failure remained localized at the interface. The observed interface traces and bond stress–slip curves indicate that curvature influenced not only the peak bond stress but also the post-peak degradation and residual stage response.
2. Within the tested range, peak, degraded stage, and residual bond stresses increased with steel tube curvature. As *K* increased from 0 to 0.02, the peak bond stress increased from 0.236 to 0.644 MPa, corresponding to an increase of 172.9%; the degraded stage characteristic stress increased from 0.150 to 0.400 MPa, an increase of 166.7%; and the residual bond stress increased from 0.113 to 0.317 MPa, an increase of 180.5%. The corresponding peak slip increased from 0.600 to 1.375 mm. In addition, the characteristic slip at the onset of the residual stage increased from 2.696 to 4.630 mm. These results indicate that increasing curvature enhanced both interfacial bond resistance and deformation capacity and extended the slip range before the response entered the residual stage.
3. Within the investigated range, increasing steel tube curvature was associated with higher peak and residual bond stresses, a more gradual post-peak degradation, and larger characteristic slips. Based on the experimental results, quantitative relationships were established between steel tube curvature and the characteristic stresses and slips at the peak, degradation, and residual stages. These curvature-dependent relationships were further incorporated into the piecewise bond–slip model, enabling the model to represent the differences in interfacial response among the investigated curvature levels. For the calibration data, the fitted relationships reproduced the measured bond–slip response with R^2^ ≥ 0.96 and a mean fitting error below 4.1%, indicating satisfactory agreement within the investigated curvature range.
4. The mechanism underlying the curvature-induced enhancement of interfacial bond behavior is identified. Increasing steel tube curvature improves the contact configuration between the steel tube and the ECC matrix, thereby increasing interfacial contact pressure and enhancing mechanical interlock and frictional resistance. The combined action of these effects restrains the initiation and propagation of interfacial bond damage and delays bond stress degradation. Consequently, the modified interface exhibits higher peak bond strength, more gradual stress degradation, and satisfactory residual bond resistance, leading to improved composite action and deformation capacity at the ECC–steel tube interface.

## Figures and Tables

**Figure 1:**
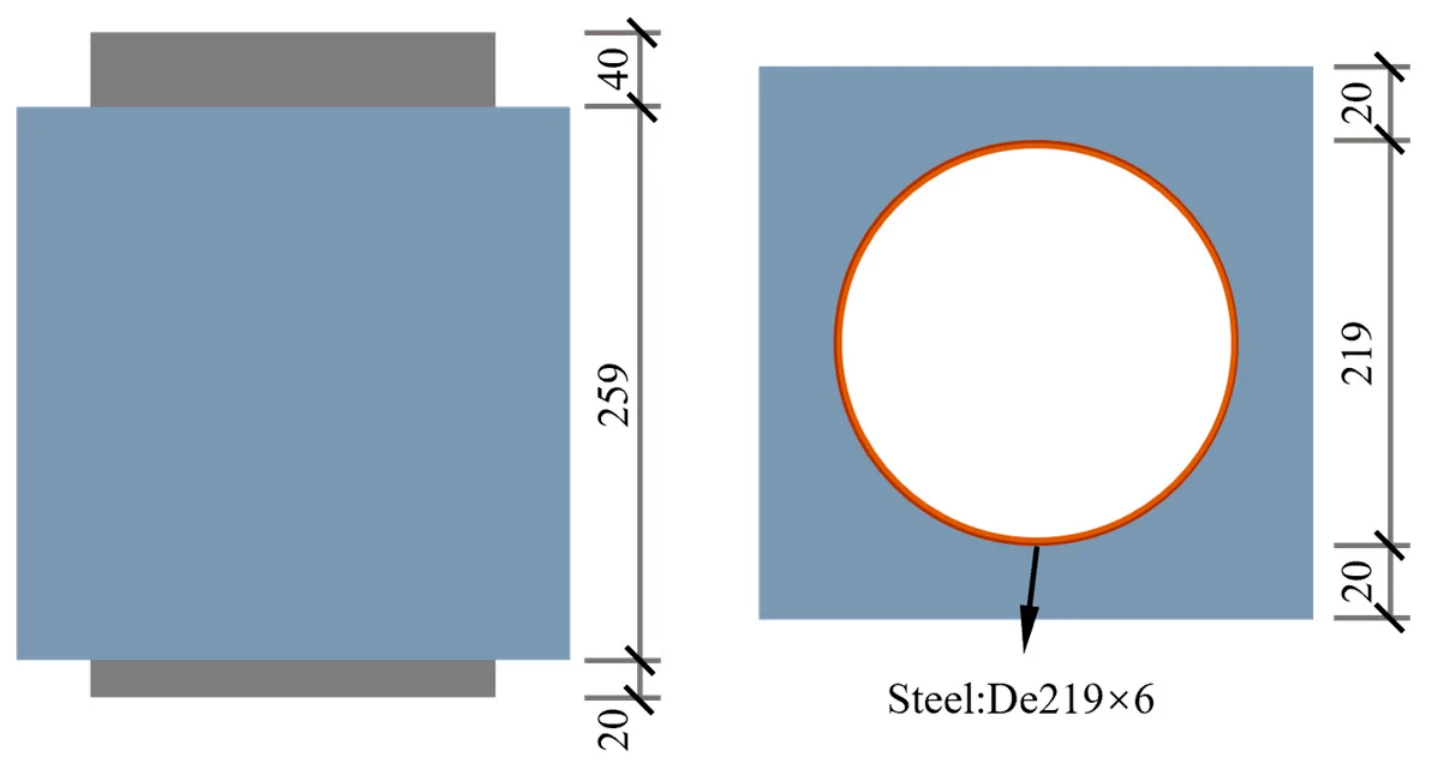
Specimen geometry and dimensions.

**Figure 2:**
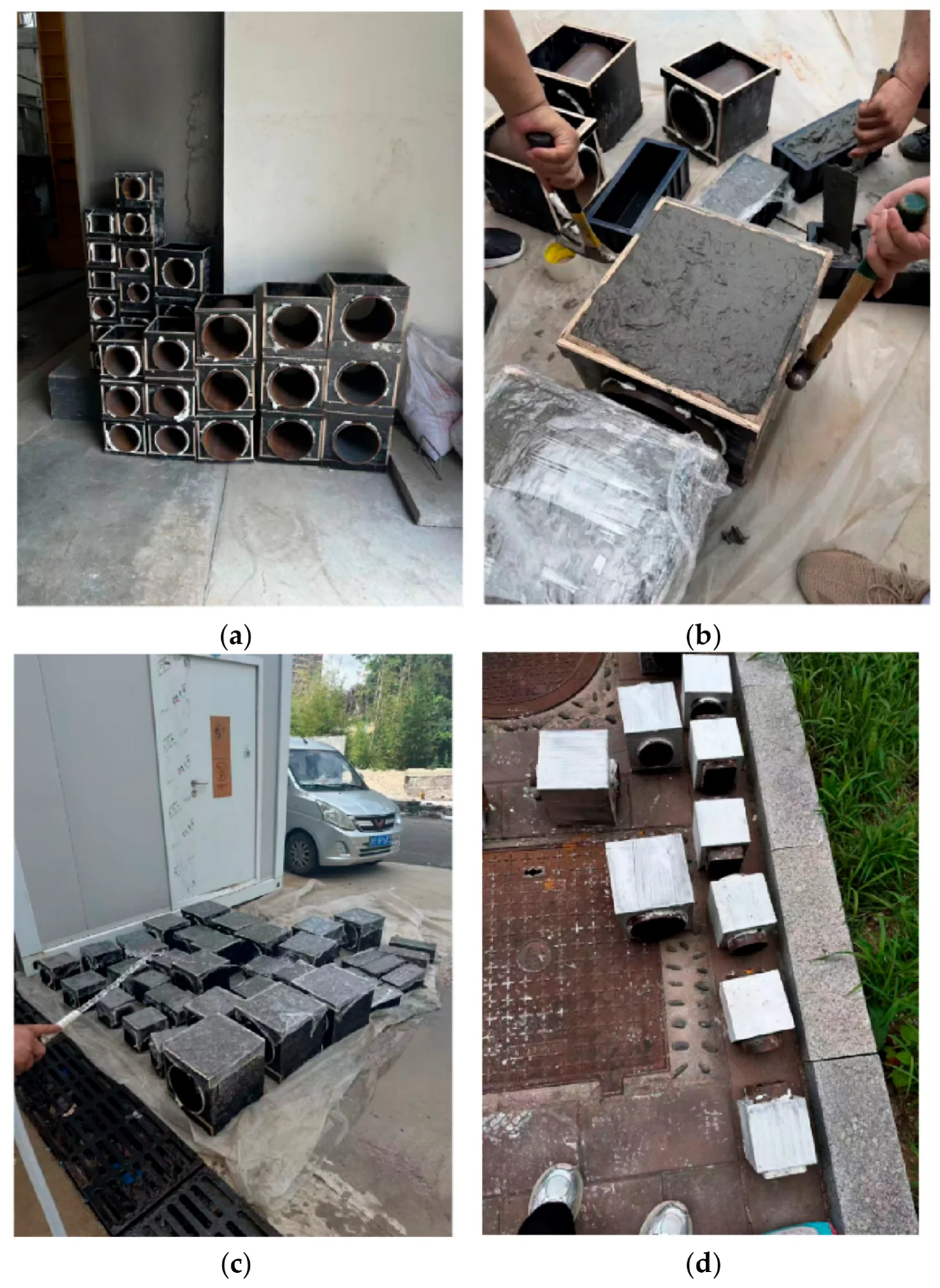
Specimen fabrication process and representative specimens before testing. (**a**) Steel tubes and formwork. (**b**) ECC casting. (**c**) Specimens after casting. (**d**) Representative specimens.

**Figure 3:**
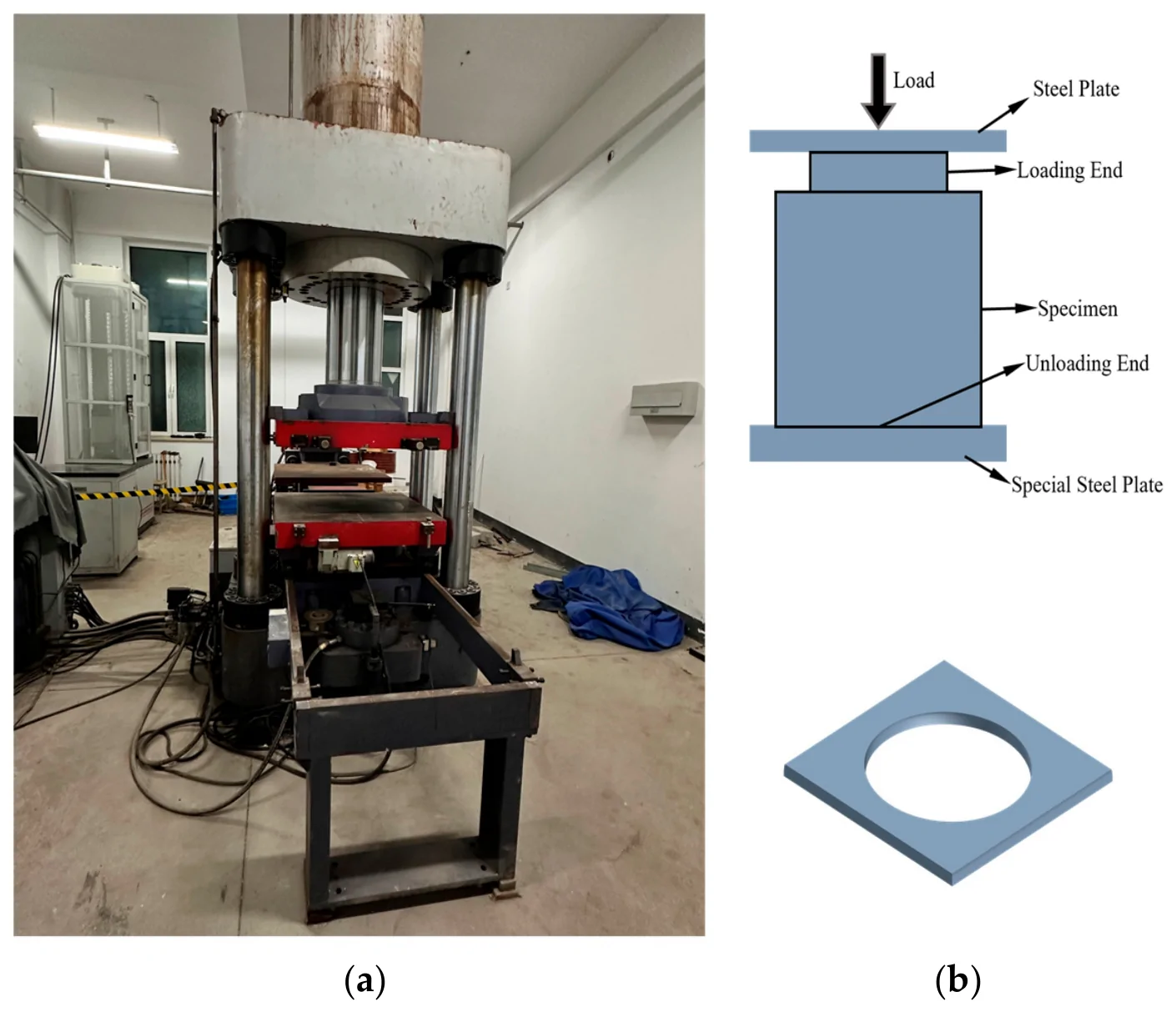
Test setup and instrumentation. (**a**) Loading device. (**b**) Schematic diagram of loading.

**Figure 4:**
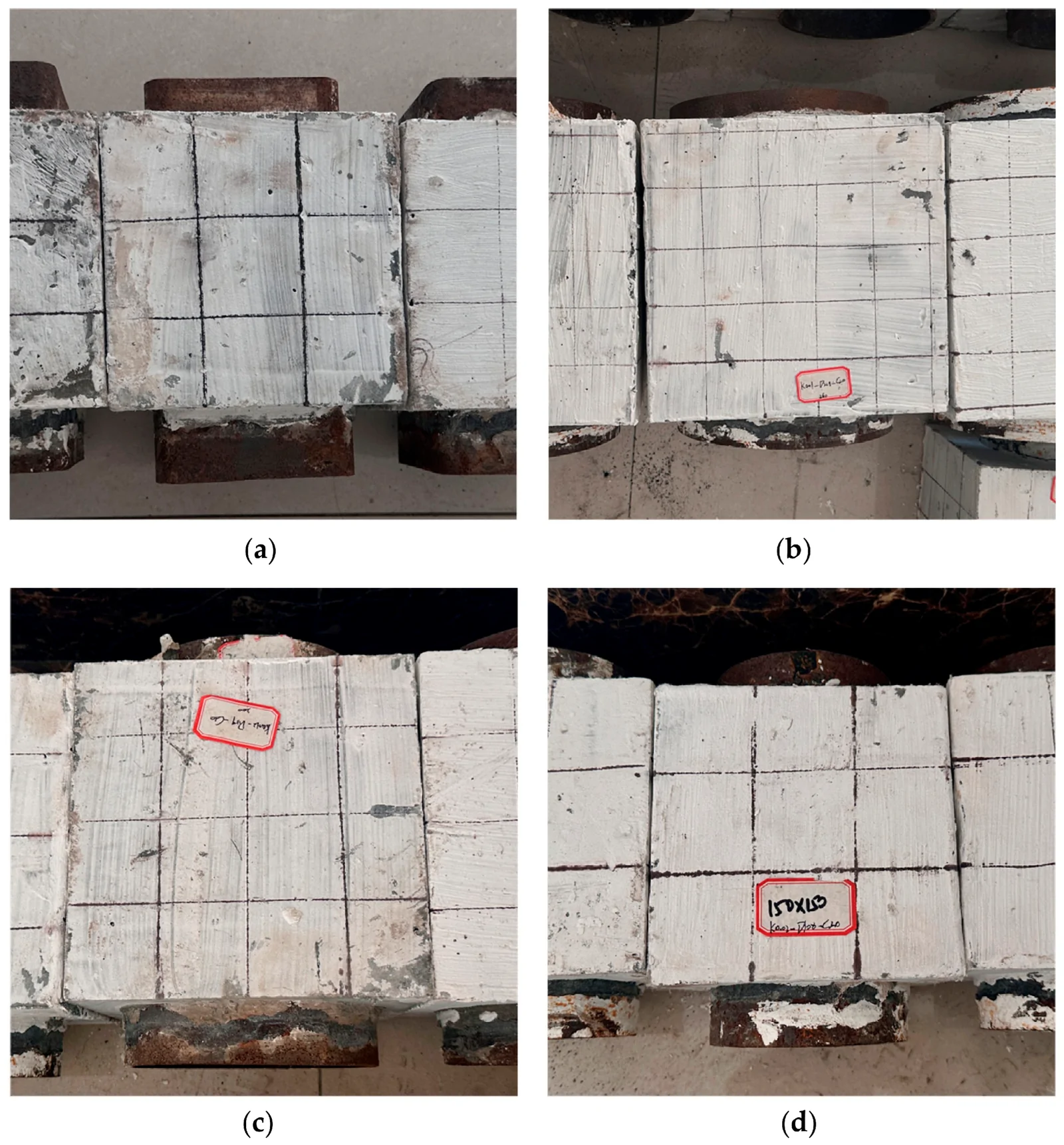
Failure appearances of the specimens. (**a**) *K*0-C20. (**b**) *K*0.01-C20. (**c**) *K*0.012-C20. (**d**) *K*0.02-C20.

**Figure 5:**
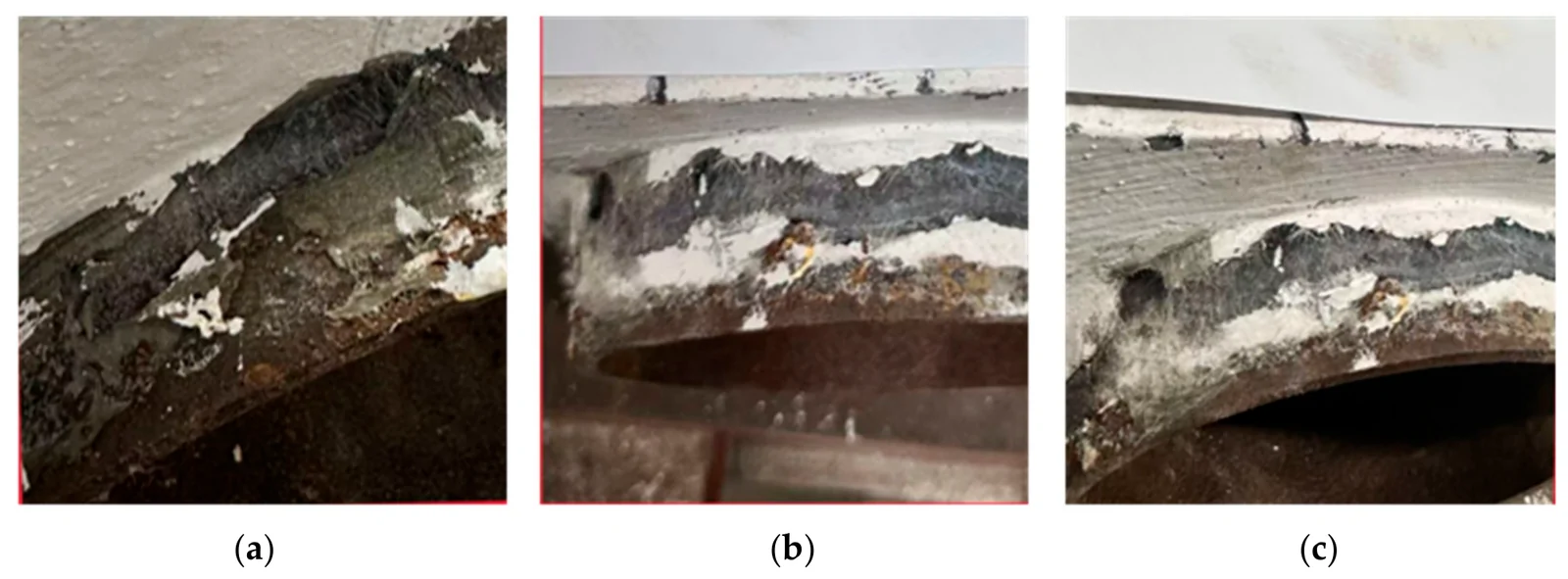
Representative interfacial damage and friction traces observed after the push-out tests. (**a**) *K*0.01-C20. (**b**) *K*0.012-C20. (**c**) *K*0.02-C20.

**Figure 6:**
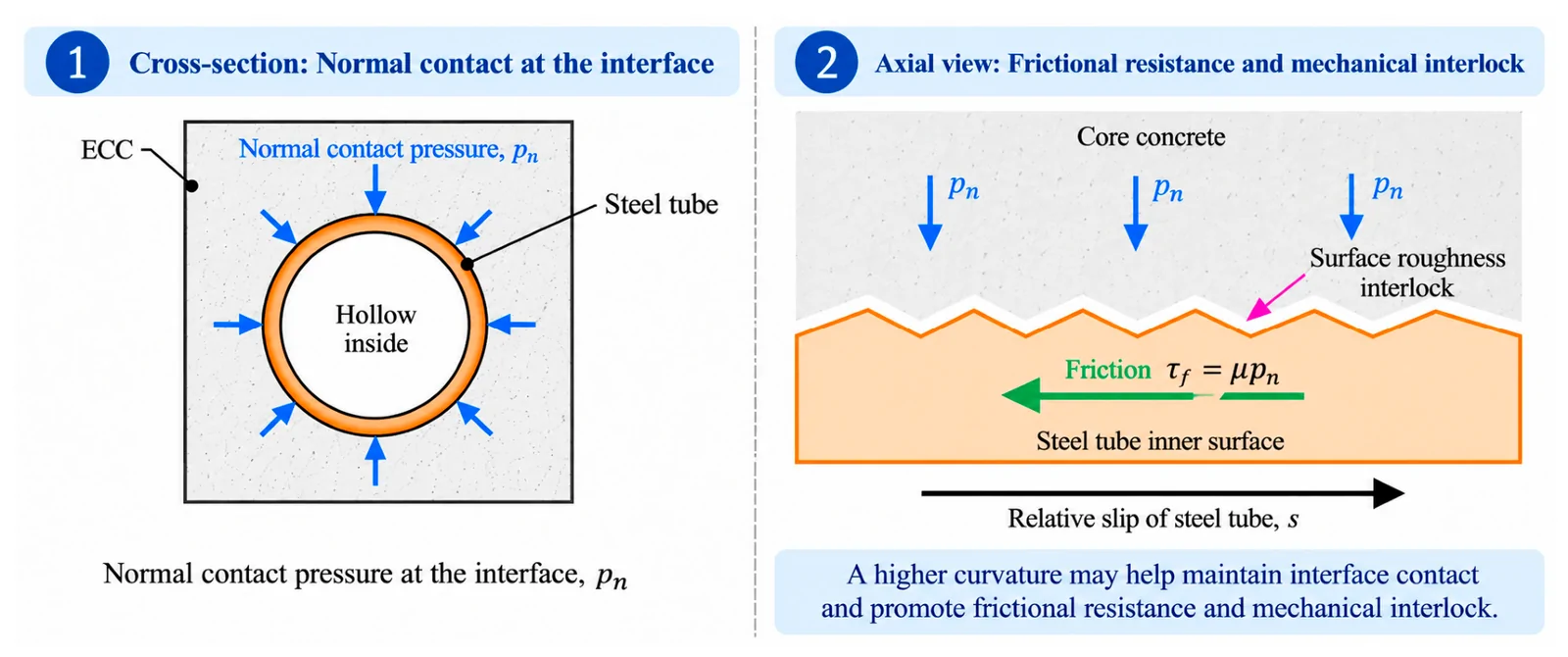
Schematic illustration of the possible interfacial load transfer mechanism affected by steel tube curvature.

**Figure 7:**
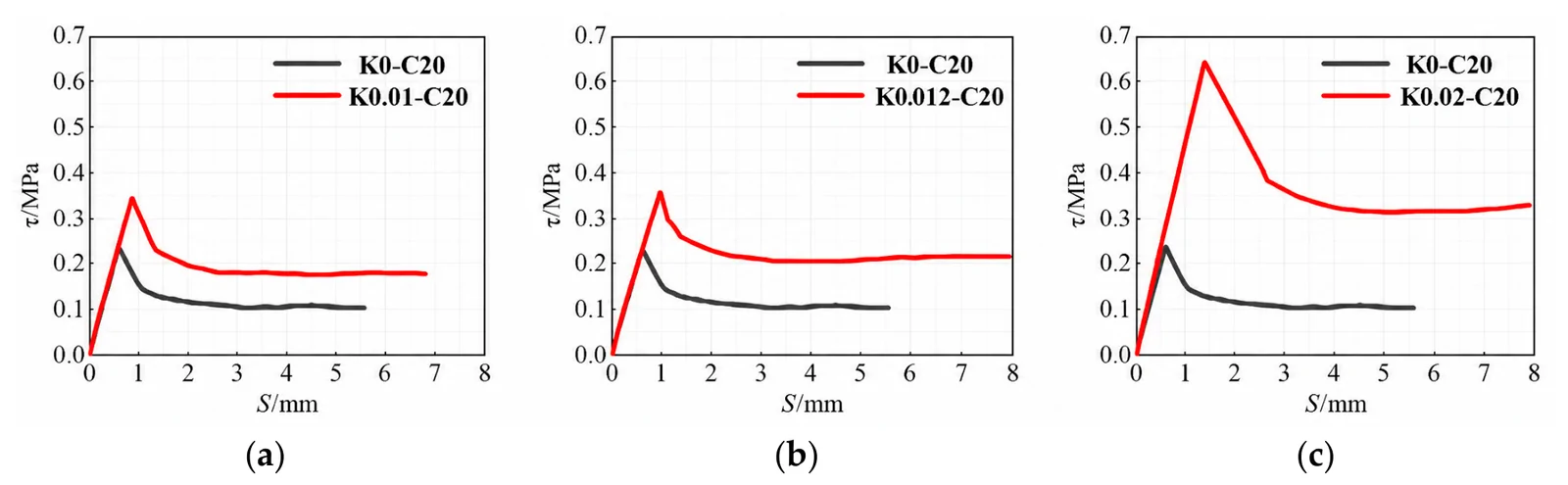
Bond stress–slip curve of the specimens. (**a**) *K*0.01-C20. (**b**) *K*0.012-C20. (**c**) *K*0.02-C20.

**Figure 8:**
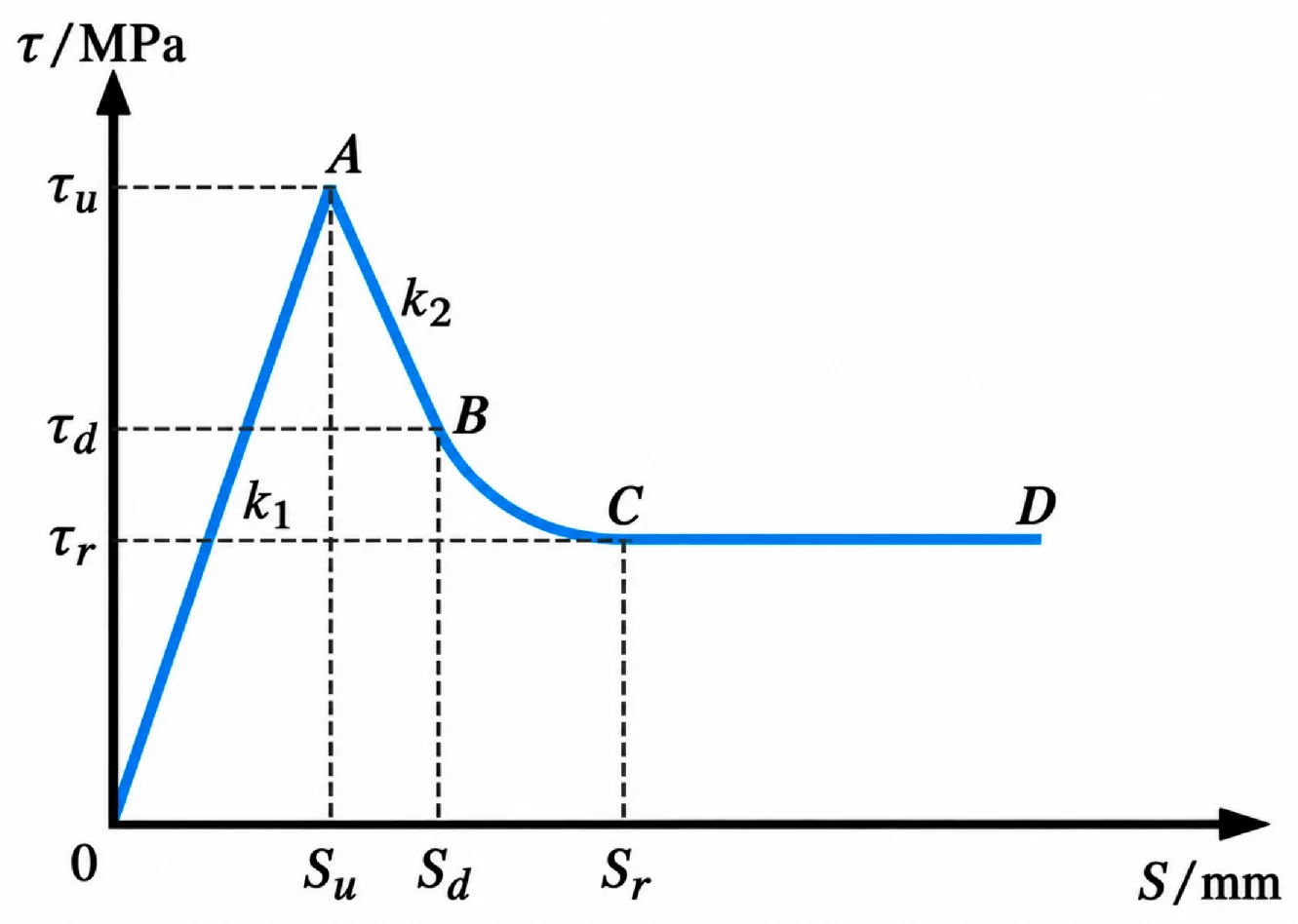
Typical bond stress–slip constitutive relationship curve.

**Figure 9:**
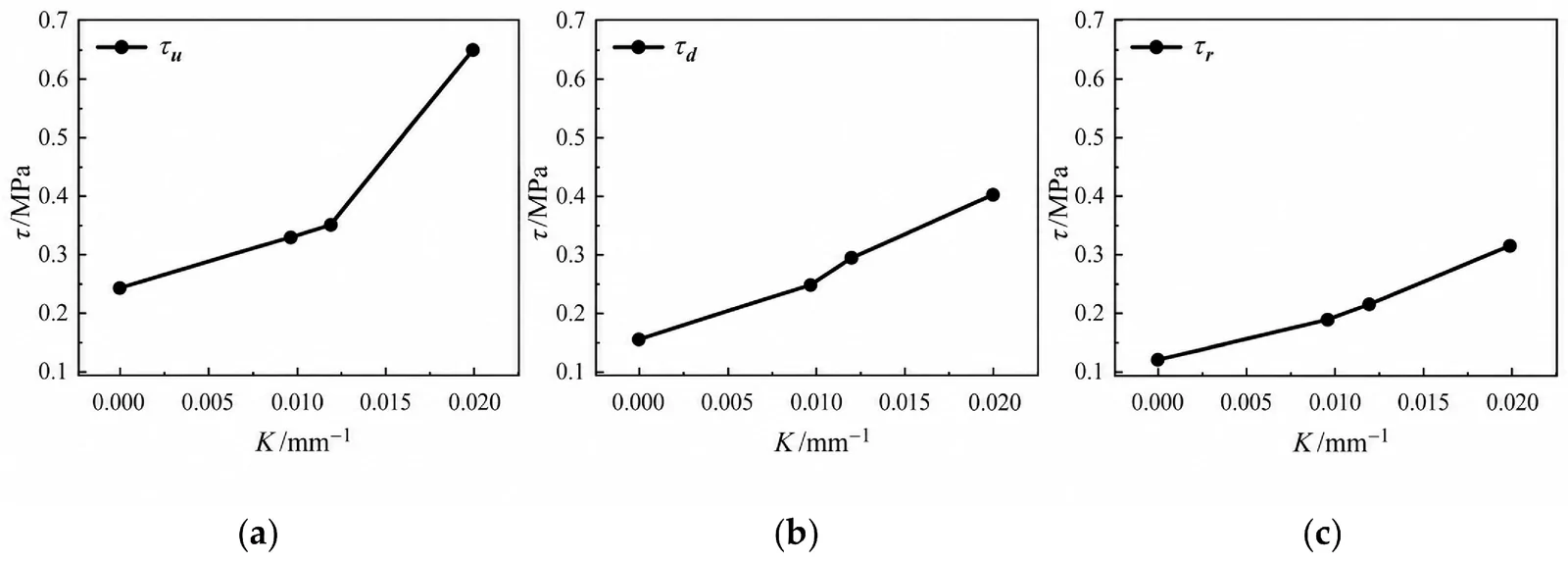
Effect of steel tube curvature on bond stress. (**a**) *τ_u_-S*. (**b**) *τ_d_-S*. (**c**) *τ_r_-S*.

**Figure 10:**
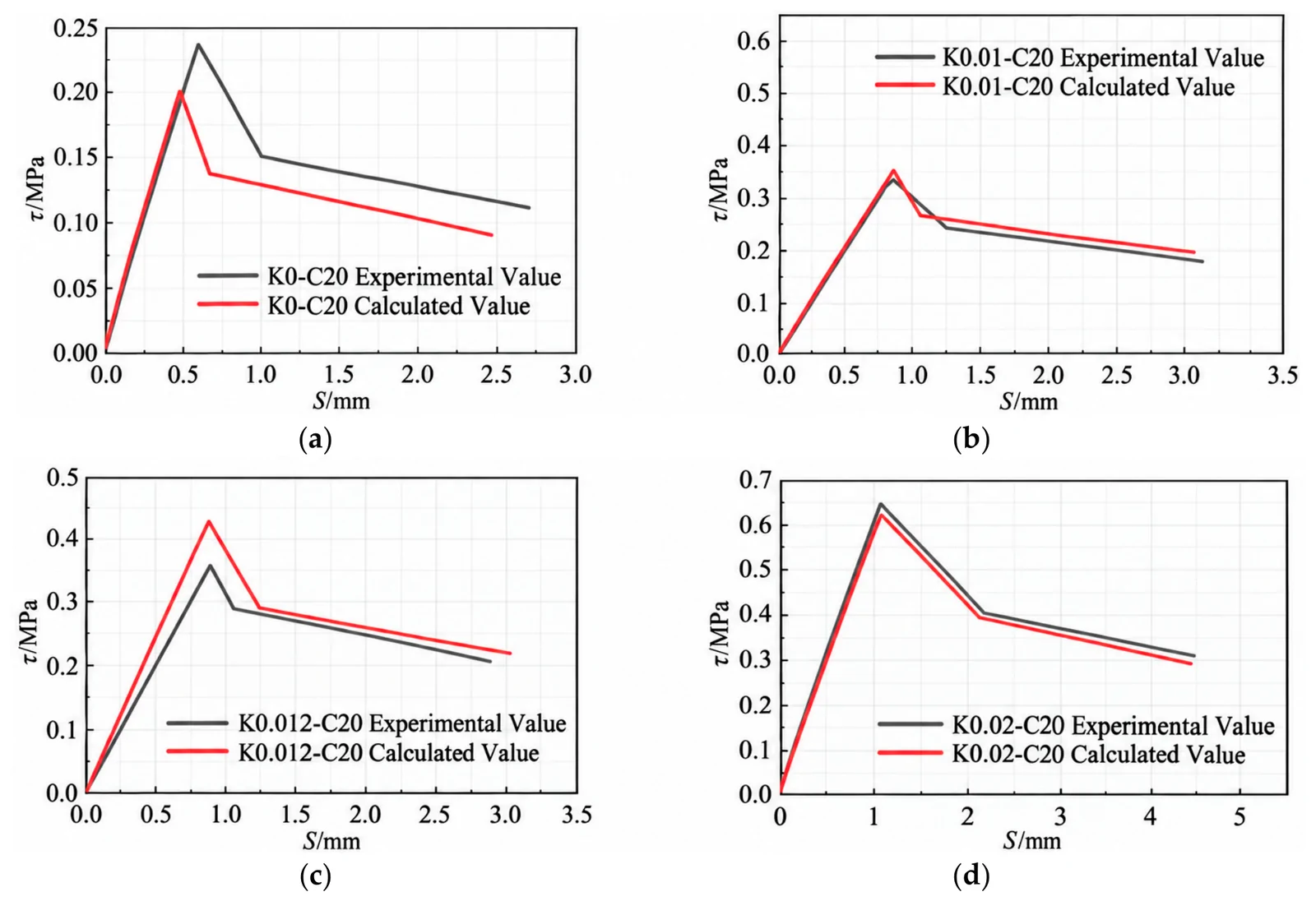
Comparison of measured and calculated bond stress–slip curves. (**a**) *K*0-C20. (**b**) *K*0.01-C20. (**c**) *K*0.012-C20. (**d**) *K*0.02-C20.

**Table 1:** Specimen parameters.

Specimen Number	Cross-Sectional Dimensions (mm)	Curvature *K*	Protective Layer Thickness C (mm)
*K*0.02-C20	148 × 148	0.02	20
*K*0.012-C20	199 × 199	0.012	20
*K*0.01-C20	259 × 259	0.01	20
*K*0.0-C20	140 × 140	0.00	20

**Table 2:** Mix proportions of ECC and performance parameters of PP fibers.

Mass Proportion	Length (mm)	Diameter (μm)	Tensile Strength (MPa)	Elastic Modulus (GPa)
Dry mix 1		-	-	-
Water 0.25		-	-	-
PP fibers 0.0125	12	30	≥300	≥3.5

**Table 3:** Mechanical properties of steel tubes.

Steel Tube Type	Wall Thickness (mm)	Tensile Strength (MPa)	Yield Strength (MPa)	Elongation After Fracture
Q235 seamless steel tube	6	375~500	235	≥26%

**Table 4:** Experimental results.

Specimen Number	*P_u_* (KN)	*P_d_* (KN)	*P_r_* (KN)	*S_u_* (mm)	*S_d_* (mm)	*S_r_* (mm)	*τ_u_* (MPa)	*τ_d_* (MPa)	*τ_r_* (MPa)
*K*0.02-C20	32.306	20.055	15.889	1.375	2.600	4.630	0.644	0.400	0.317
*K*0.012-C20	35.742	29.092	20.666	0.970	1.115	3.095	0.360	0.293	0.208
*K*0.01-C20	61.043	43.167	32.403	0.843	1.254	3.031	0.343	0.242	0.182
*K*0.0-C20	13.223	8.400	6.300	0.600	1.007	2.696	0.236	0.150	0.113

Note: *P_u_* denotes the peak load; *P_d_* denotes the load at the end of the initial post-peak decrease; *P_r_* denotes the residual load; *S_u_* denotes the slip corresponding to *P_u_*; *Sd* denotes the slip corresponding to *P_d_*; *S_r_* denotes the slip corresponding to *P_r_*; *τ_u_* denotes the bond stress corresponding to *P_u_*; *τ_d_* denotes the bond stress corresponding to *P_d_*; *τ_r_* denotes the bond stress corresponding to *P_r_*.

**Table 5:** Variability of the characteristic bond–slip parameters among repeated specimens.

Specimen Group	*SD_τu_* (MPa)	*SD_τd_* (MPa)	*SD_τr_* (MPa)	*SD_Su_* (mm)	*SD_Sd_* (mm)	*SD_Sr_* (mm)
*K*0-C20	0.0180	0.0110	0.0085	0.0680	0.1150	0.3429
*K*0.01-C20	0.0270	0.0173	0.0135	0.0742	0.1450	0.3783
*K*0.012-C20	0.0280	0.0220	0.0143	0.1308	0.1200	0.2687
*K*0.02-C20	0.0480	0.0280	0.0230	0.1650	0.2298	0.5975

Note: *SD* denotes the standard deviation calculated from the repeated specimens within each curvature group. The characteristic parameters were identified according to the definitions adopted in Figure 8 and Table 4.

**Table 6:** Calculated bond stress and slip parameters.

Specimen Number	$\tau_{u}^{\prime }$ (MPa)	$\tau_{d}^{\prime }$ (MPa)	$\tau_{r}^{\prime }$ (MPa)	$S_{u}^{\prime }$ (mm)	$S_{d}^{\prime }$ (mm)	$S_{r}^{\prime }$ (mm)
*K*0.02-C20	0.583	0.390	0.300	1.312	2.204	4.243
*K*0.012-C20	0.425	0.290	0.220	1.005	1.606	3.502
*K*0.01-C20	0.386	0.265	0.200	0.928	1.457	3.317
*K*0.0-C20	0.188	0.140	0.099	0.544	0.709	2.390

**Table 7:** Ratios of calculated to measured values.

Specimen Number	$\frac{\tau_{u}^{\prime }}{\tau_{u}}$ (MPa)	$\frac{\tau_{d}^{\prime }}{\tau_{d}}$ (MPa)	$\frac{\tau_{r}^{\prime }}{\tau_{r}}$ (MPa)	$\frac{S_{u}^{\prime }}{S_{u}}$ (mm)	$\frac{S_{d}^{\prime }}{S_{d}}$ (mm)	$\frac{S_{r}^{\prime }}{S_{r}}$ (mm)
*K*0.02-C20	0.905	0.975	0.946	0.954	0.848	0.914
*K*0.012-C20	1.181	0.990	1.058	1.036	1.440	1.132
*K*0.01-C20	1.125	1.095	1.099	1.101	1.162	1.094
*K*0.0-C20	0.797	0.933	0.876	0.907	0.704	0.884

## Data Availability

The original contributions presented in this study are included in the article. Further inquiries can be directed to the corresponding author.
